# A Piezoresponse Force Microscopy Study of Ca*_x_*Ba_1−*x*_Nb_2_O_6_ Single Crystals

**DOI:** 10.3390/ma10091032

**Published:** 2017-09-05

**Authors:** Vladimir V. Shvartsman, Danka Gobeljic, Jan Dec, Doru C. Lupascu

**Affiliations:** 1 Institute for Materials Science and Center for Nanointegration Duisburg-Essen (CENIDE), University of Duisburg-Essen, 45141 Essen, Germany; danka.gobeljic@uni-due.de (D.G.); doru.lupascu@uni-due.de (D.C.L.); 2 Institute of Materials Science, University of Silesia, 40-007 Katowice, Poland; jan.dec@us.edu.pl

**Keywords:** scanning probe microscopy, ferroics, domains, piezoresponse force microscopy, relaxors, (Ca, Ba) Nb_2_O_6_, tungsten bronze

## Abstract

Polar structures of Ca*_x_*Ba_1−*x*_Nb_2_O_6_ (CBN100*x*) single crystals were investigated using piezoresponse force microscopy. Increasing Ca content results in decreasing domain size and enhancement of the polar disorder. For the composition with *x* = 0.32 the characteristic domain size is similar to that reported for relaxor Sr_0.61_Ba_0.39_Nb_2_O_6_ (SBN61). However, decay of an artificial macroscopic domain in CBN32 takes place below the macroscopic transition temperature, contrary to SBN61, where random fields stabilize it above the transition temperature. We can conclude that CBN with 0.26 ≤ *x* ≤ 0.32 does not display classical relaxor behavior and might be considered as a disordered ferroelectric.

## 1. Introduction

Relaxor ferroelectrics with the tetragonal tungsten bronze (TTB) structure have attracted significant attention as the lead-free alternative to well-known PbMg_1/3_Nb_2/3_O_3_-based compounds [1]. In particular, the Sr*_x_*Ba_1−*x*_Nb_2_O_6_ (SBN) system has been intensively studied due to promising pyroelectric, electrooptic, and photorefractive properties [2,3,4]. In this system, the cross-over from the ferroelectric to relaxor behavior occurs upon increasing Sr content above *x* = 0.61 [5,6]. Recently, the Ca*_x_*Ba_1−*x*_Nb_2_O_6_ (CBN100*x*) system, the Ca counterpart of SBN, has attracted interest primarily due to its high Curie temperature, T_C_ = 200–270 °C, i.e., about 200 °C higher than that of SBN [7,8]. Contrary to SBN, CBN single crystals show a reduced composition existence region between 0.20 < *x* < 0.40 because the smaller Ca cation can only occupy the A1 position in the TTB structure. The properties of CBN are still a matter of discussion. Based on the results of thermal expansion measurements and resonant ultrasound spectroscopy, CBN28 was classified as a relaxor [9]. Brillouin scattering experiments indicated the appearance of characteristics of the relaxors polar nanoregions at temperatures much higher than the ferroelectric transition temperature [10]. It was also argued that increasing the Ca content results in more pronounced relaxor behavior [11]. However, contrary to SBN relaxors, the polarization relaxation time of CBN28 shows a critical slowing down towards the Curie temperature, reflecting a weak first-order phase transition [10]. Moreover, CBN crystals do not show the frequency dispersion of the temperature of the maximum dielectric permittivity that is typical for relaxors [7,12]. It was also suggested that the relaxor-like behavior of the CBN crystals appears as a result of an external action (e.g., an alternating electric field) and does not exist in the as-grown state [12].

Important information about relaxor or ferroelectric behavior of CBN crystals can be obtained by investigation of their polar structures. Scanning and transmission electron microscopy studies of the (001) cut of CBN28 crystals revealed fine 180° domain patterns with domain sizes in the range of 30–200 nm [7,13]. These methods, however, are destructive and allow for observation of static patterns only. Non-destructive but indirect investigations of the evolution of domain structures in the vicinity of the Curie temperature of CBN28 were performed via second harmonic generation *k*-space spectroscopy [14]. A nonuniformity of the ferroelectric phase transition for differently sized polar structures was revealed, which resembles the diffuse phase transition model proposed by Smolenskii [15].

Non-destructive direct studies of the evolution of polar structures are possible using piezoresponse force microscopy (PFM) [16]. PFM is based on the detection of local piezoelectric deformation induced by an *ac* voltage applied between a sample and a nanometer sharp tip. The phase of the first harmonic of this deformation provides information about the local polarization orientation, while its amplitude is proportional to the local piezoelectric coefficient. PFM has been successfully applied to the investigation of SBN single crystals, where a cross-over from ferroelectric to relaxor behavior on increasing of Sr concentration was accompanied by an enhanced polar disorder [17]. Moreover, PFM allowed for the direct observation of static polar nanoregions (PNRs) above the transition temperature, which had been predicted for relaxors [17].

In this article, we report on piezoresponse force microscopy (PFM) studies of CBN single crystals. As in the case of SBN, a diminishment of domain size was found in CBN as the Ca/Ba ratio increases. The characteristic size of nanodomains in CBN32 is comparable with that in SBN61. However, contrary to relaxor SBN61, the depolarization of a macroscopic domain in CBN31 occurs below the transition temperature, but not above it.

## 2. Results and Discussion

**Figure 1:**
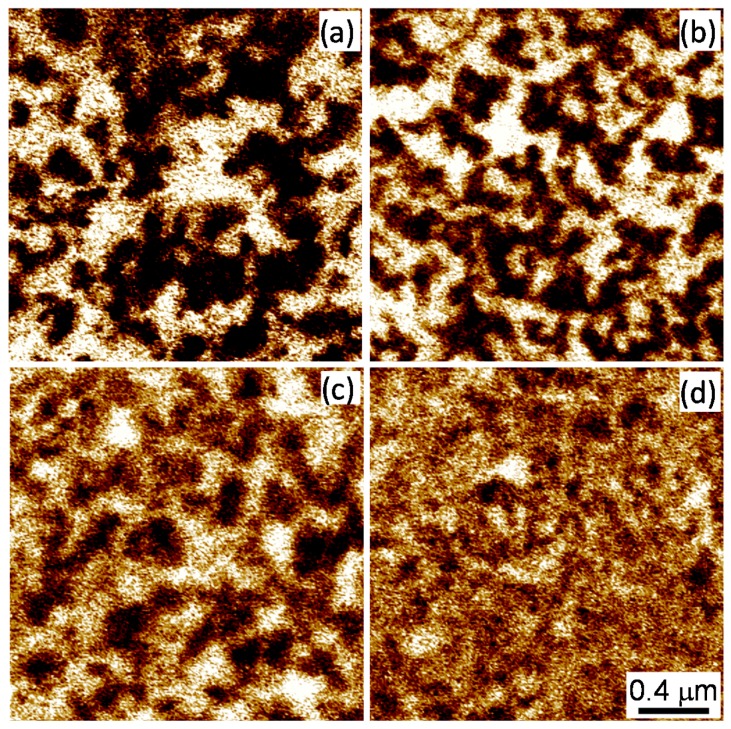
Vertical PFM images of (001) cuts of CBN26 (**a**); CBN28 (**b**); CBN30 (**c**); and CBN32 (**d**) single crystals taken at room temperature.

Figure 1 shows vertical PFM images of (001) cut CBN single crystals with different compositions taken at room temperature. The vertical PFM signal is sensitive to the component of polarization normal to the sample surface. Dark and bright regions correspond to domains with polarization oriented up and down, respectively. A brownish contrast corresponds to regions with zero (or very small) piezoresponse. According to the results of dielectric measurements, even the composition with the largest Ca content (CBN32) has a transition to the ferroelectric state at about 470 K [12]. Thus, all samples are well below the transition temperature. Nevertheless, we can clearly see a variation of the domain structure as Ca content increases. Namely, the domains become smaller, domain boundaries appear more jagged, and the relative area of regions showing a weak piezoresponse increases.

**Figure 2:**
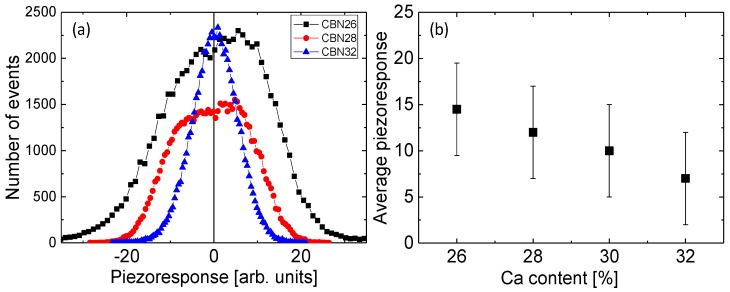
(**a**) Histograms of the PFM images shown in Figure 1; (**b**) dependence of the average piezoresponse on Ca concentration.

Figure 2a shows histograms of the piezoresponse inside the studied regions. Since only two polarization directions (up and down) are expected, we can estimate an average piezoresponse that provides us with a measure of the local polarization from the positions of the peaks on these histograms. Unfortunately, no distinct peaks can be observed, even for the CBN26 sample. Instead, a broad maximum is seen around zero piezoresponse, which becomes narrower as Ca content increases. A deconvolution of this broad maximum into three peaks (positive, negative, and zero piezoresponse) is possible. Figure 2b shows the concentration dependence of the local piezoresponse estimated from the positions of the “positive” and “negative “maxima. One can see that the piezoresponse drops as Ca content increases.

**Figure 3:**
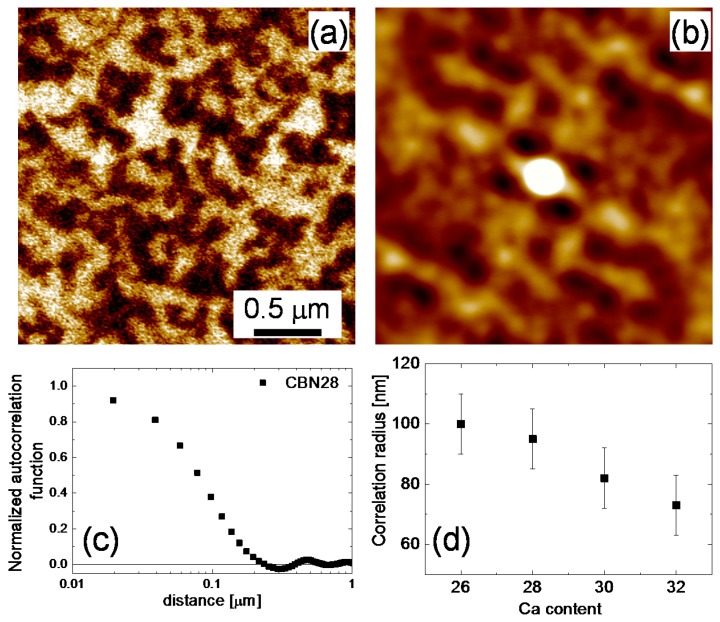
(**a**) Vertical PFM image of CBN28 single crystal; (**b**) corresponding autocorrelation image; (**c**) autocorrelation function averaged over in-plane directions; (**d**) dependence of the correlation radius on Ca concentration.

To evaluate the size of the domains, we performed an autocorrelation analysis that was successfully applied for the investigation of nanopolar structures in relaxor ferroelectrics [16]. Autocorrelation images, *C*(*r*_1_,*r*_2_), (Figure 3b) were obtained from the original PFM images via the following transformation:

(1) $$C(r_{1},r_{2})=\sum_{x,y}D(x,y)D(x+r_{1},y+r_{2})$$

where *D*(*x*,*y*) is the value of the piezoresponse signal at a point with coordinates (*x*,*y*). From the shape of the autocorrelation function, information about the symmetry and regularity of the domain structure can be obtained. In particular, the width of the central peak in the *C*(*r*_1_,*r*_2_) map is a measure of the polarization correlation radius, i.e., domain size. To evaluate it, the autocorrelation function was averaged over all in-plane directions (Figure 3c) and then approximated by the function:

(2) $$<C(r)>=\sigma^{2}\exp\left[-{\left(r/<\xi >\right)}^{2h}\right].$$

Here, *r* is the distance from the central peak, <*ξ*> is the average correlation radius, and the exponent *h* (0 < *h* < 1) is a measure of the roughness of the “polarization interface” [16]. We observed that increasing Ca content in CBN results in a decrease in the correlation radius from 100 (CBN26) to 70 nm (CBN32) (Figure 3d).

**Figure 4:**
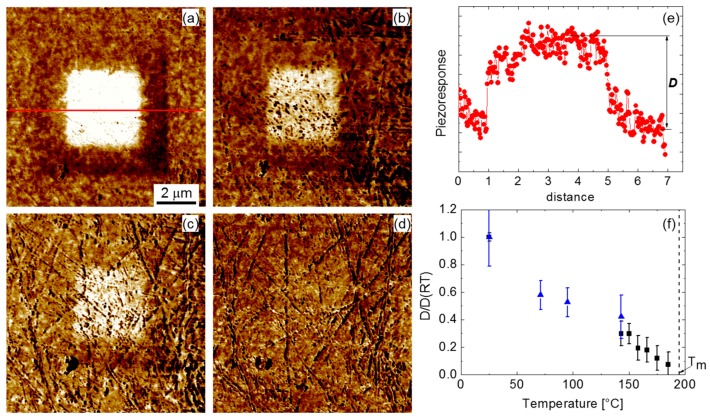
(**a**–**d**) Vertical PFM images of the (001) cut of the CBN32 single crystal after central part was scanned under dc bias −40 V: (**a**) room temperature, (**b**) 95 °C, (**c**) 140 °C, and (**d**) 185 °C; (**e**) An example of a cross section of the poled area; (**f**) Temperature dependence of the piezoresponse inside the poled area normalized to the value at room temperature.

To investigate the evolution of the polar structure in the vicinity of the phase transition temperature, we chose the CBN32 single crystal that shows the lowest transition temperature (around 195 °C) and that presumably is a relaxor. We prepared a “macroscopic” domain by scanning an area of 5 × 5 µm^2^ by applying a *dc* voltage of −40 V. As a result, a uniformly poled area appeared (Figure 4a). On heating, both a gradually decreasing PFM contrast inside the area and its decay were observed. To evaluate the temperature evolution of the piezoresponse inside this domain, we analyzed averaged cross-sections of the PFM images. Figure 4f shows the temperature dependence of the piezoresponse normalized to its value at room temperature. We observed, first, a substantial decay (about 40%) of the piezoresponse between room temperature and 70 °C. This can be partially related to the temporal relaxation of the induced domain. Isothermal experiments indicated that the piezoresponse decays by about 15% during the first two hours after poling and by 25% in the course of three days. Between 70 and 140 °C, the decay of the piezoresponse is less significant. However, at approximately 150 °C, it starts to decay again. Finally, at around 185 °C (i.e., slightly below the transition temperature), the macroscopic domain disappears completely.

**Figure 5:**
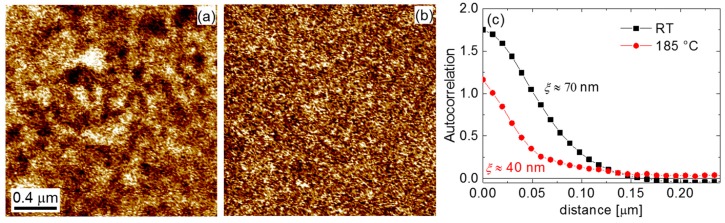
Vertical PFM images of the (001) cut of the CBN32 single crystal at (**a**) room temperature and (**b**) 185 °C; (**c**) corresponding autocorrelation functions averaged over in-plane directions.

The investigation of the non-poled area shows that nanodomains can be still observed at this critical temperature (Figure 5). While they are much smaller and show a weaker response than those at room temperature, they definitely can be distinguished from the noise background. The autocorrelation analysis shows that the corresponding correlation radius is about 40 nm.

Thus, we observed that the induced “macrodomain” disappears on approaching the transition temperature, while the nanodomains still exist. This behavior is in contrast to one reported for Sr_0.61_Ba_0.39_Nb_2_O_6_ single crystals [18]. In the latter case, the poled state was not deleted even above the phase transition temperature. The authors attributed its stability to the fact that the decay into the paraelectric phase is hampered by local pinning forces of the quenched electrical random fields counteracting the establishment of the paraelectric disorder [18]. Seemingly, in the case of CBN, the effect of the random fields is much weaker. Indeed, in CBN Ca^2+^ ions are restricted to the square A1 sites, whereas the pentagonal A2 sites are nearly fully occupied by Ba^2+^ exclusively [14]. In contrast, in SBN, Sr^2+^ is equally distributed between A1 and A2; additionally, A2 is less than 50% occupied by barium. Therefore, less structural disorder and, correspondingly, weaker random fields are expected for CBN as compared to SBN [10]. We also observed that increasing Ca content results in a larger polar disorder, which manifests itself in a finer and less compact domain structure. Similarly, as was suggested for SBN [19], we can consider that compositions with larger amounts of Ca have more structural and related polar disorders. However, a limited compositional range of CBN hampers the development of “canonical” relaxor behavior that is observed in SBN with Sr content more than 61%. Even though the nanodomains have a similar size in the ferroelectric state of SBN61 and CBN32, the latter compound does not show the characterictic relaxor features. Indeed, the temperature dependence of the dielectric permittivity of CBN crystals does not show the typical for relaxors dispersion of the peak position [12]. We suggest classifying CBN32 as a disordered ferroelectric, where the structural disorder is strong enough to prevent the formation of large domains but rather weak to induce polar nanoregions and related relaxor behavior [5].

## 3. Materials and Methods

The studied Ca*_x_*Ba_1−*x*_Nb_2_O_6_ single crystals, where *x* = 0.26−0.32, were grown by the Czochralski method [20]. Experiments were done on platelet-shaped samples (with a thickness of ~0.5 mm) with optically polished faces. The (001) cut perpendicular to the polarization direction was investigated. PFM studies were performed using a commercial atomic force microscope MFP-3D (Asylum Research, Santa Barbara, CA, USA). Ti/Ir-coated tip-cantilevers, Asylelec 01 (Asylum Research, Santa Barbara, CA, USA), with a spring constant *k* = 2 N/m were used. The measurements were performed by applying an *ac* voltage with an amplitude *U_ac_* = 5–10 V and a frequency *f* = 50 kHz. A heating stage was used to conduct PFM measurements between room temperature and 185 °C.

## 4. Conclusions

In CBN single crystals, increasing Ca content results in both decreasing domain size and local polarization. In general, in compositions with more Ca content, the polar disorder becomes more pronounced. The characteristic nanodomain size of the CBN32 single crystal is similar to that of relaxor SBN61. However, the decay of an artificial macroscopic domain (depolarization) in CBN32 takes place below the temperature of the maximum of the dielectric permittivity, but not above it like in the case of SBN61. Based upon the obtained data and earlier dielectric permittivity data [12], we consider the CBN crystals as disordered ferroelectrics. Here, structural disorder prevents the formation of macroscopic domains, even in bulk materials. Related random fields are not sufficient to stabilize PNRs in the paraelectric phase, and relaxor behavior does not arise.
